# Differences in Acellular Reactive Oxygen Species (ROS) Generation by E-Cigarettes Containing Synthetic Nicotine and Tobacco-Derived Nicotine

**DOI:** 10.3390/toxics10030134

**Published:** 2022-03-11

**Authors:** Shaiesh Yogeswaran, Irfan Rahman

**Affiliations:** Department of Environmental Medicine, University of Rochester Medical Center, Box 850, 601 Elmwood Avenue, Rochester, NY 14642, USA; shaiesh_yogeswaran@urmc.rochester.edu

**Keywords:** tobacco-free nicotine (TFN), synthetic nicotine, tobacco-derived nicotine, vape-bar, electronic nicotine delivery systems, reactive oxygen species (ROS)

## Abstract

Electronic nicotine delivery systems (ENDS) containing synthetic nicotine have yet to be classified as tobacco products; consequently, there is ambiguity over whether Food and Drug Administration (FDA) regulatory authority can be extended to include tobacco-free nicotine (TFN) e-cigarettes. In recent years, a more significant number of e-cigarette companies have been manufacturing TFN-containing e-cigarettes and e-liquids to circumvent FDA regulations. While studies have shown that aerosols generated from tobacco-derived nicotine-containing e-cigarettes contain significant reactive oxygen species (ROS) levels, no comparison studies have been conducted using TFN e-cigarettes. This study uses a single puff aerosol generator to aerosolize TFN and tobacco-derived nicotine-containing vape products and subsequently involves semi-quantifying the ROS generated by these vape products in H_2_O_2_ equivalents. We found that the differences between ROS levels generated from TFN and tobacco-derived nicotine-containing vape products vary by flavor. TFN tobacco flavored and fruit flavored products are more toxic in terms of ROS generation than menthol/ice and drink/beverage flavored products using TFN. Our study provides further insight into understanding how flavoring agents used in vape products impact ROS generation from e-cigarettes differently in TFN e-cigarettes than e-cigarettes using tobacco-derived nicotine.

## 1. Introduction

Based on data from the 2021 National Youth Tobacco Survey (NYTS), a report published in the Morbidity and Mortality Weekly Report estimated 11.3% (1.72 million) of high school students and an estimated 2.8% (320,000) of middle school students currently use e-cigarettes [1]. E-cigarette aerosols contain numerous toxic chemicals, including acrolein, formaldehyde, and acetaldehyde; the latter two are known to cause lung disease and cardiovascular disease [2,3]. Previous studies have shown that aerosols generated from e-cigarette vapor contain exogenous reactive oxygen species (ROS) [4,5,6]. Additionally, studies have shown that exogenous ROS found in cigarette smoke and air pollutants can induce oxidative stress in the lungs and are the main factor in the development of chronic obstructive pulmonary disease (COPD) [7].

The 2021 NYTS found that out of all youth e-cigarette users surveyed, 85% used flavored e-cigarettes [1]. Additionally, one study has shown that ROS levels within e-cigarette aerosols vary amongst different flavored e-cigarettes and e-cigarettes of differing nicotine concentrations [4]. Regarding analyses of e-cigarette sales trends, a study conducted by the Office on Smoking and Health, a part of the Center for Disease Control and Prevention (CDC), found that 98.7% of flavored e-cigarettes sold in the United States in 2015 contain nicotine [8]. Ongoing efforts to reduce youth usage of e-cigarettes include the Food and Drug Administration (FDA) extending its tobacco regulatory authority to cover electronic nicotine delivery systems (ENDS), like e-cigarettes, in 2016 [9]. In May 2016, the FDA issued the Deeming Tobacco Products to be Subject to the Federal Food, Drug, and Cosmetic Act, commonly known as the “Deeming Rule” [9]. Under the “deeming rule,” the FDA can regulate the sales of any product that contains tobacco or uses components derived from tobacco, like tobacco-derived nicotine; this includes e-cigarettes [9]. Moreover, since May 2016, the FDA has required all e-cigarette manufacturers and retailers to file premarket tobacco market applications (PMTAs) to gain permission from the agency to market their products [9]. The Center for Tobacco Products (CTP) oversees all products containing tobacco-derived nicotine; however, the FDA has not decided how to regulate synthetic nicotine-containing vape products; these products continue to remain unregulated [2,10,11]. In recent years, a more significant number of e-cigarette manufacturers have been using synthetic nicotine instead of tobacco-derived nicotine when producing e-cigarettes and e-liquids, all to bypass/evade FDA regulations [10]. Synthetic nicotine is chemically identical to nicotine from tobacco plants, with the former being made within a lab without the need of a tobacco plant [12]. In February 2021, Puff Bar, a prominent e-cigarette manufactured in the U.S., reintroduced their disposable vape-bar products, claiming them to contain synthetic nicotine and not containing tobacco or anything derived from tobacco [13]. Since Puff Bar’s synthetic nicotine-containing vape bars entered the market in April 2021, Puff Bar has become the most popular company from which disposable e-cigarettes are purchased in the U.S., the company holding 51.3% of the national disposable e-cigarette market share [13]. No studies to date have been conducted involving comparative analyses in exogenous ROS levels between aerosols generated by synthetic-nicotine-containing e-cigarettes and those by e-cigarettes containing tobacco-derived nicotine. With the substantial rise in youth usage of e-cigarettes and a more significant number of e-cigarette manufacturers producing TFN e-cigarettes, more studies examining differences in ROS levels between aerosols generated by tobacco-based nicotine and synthetic nicotine-containing e-cigarettes are needed [11]. Unlike previous studies which have analyzed the ROS concentration levels within aerosols generated by tobacco-derived nicotine-containing e-cigarettes, our study includes analyses of the acellular ROS levels generated by TFN e-cigarettes [4,5,6]. Adding to the novelty of this study, we seek to understand the role the type of salt nicotine used in e-flavored e-cigarettes (synthetic or tobacco-derived) has in altering acellular ROS levels within generated aerosols. In this study, we quantify ROS levels generated by synthetic nicotine-containing ENDS products and compare them to ROS levels generated from their flavor-specific tobacco-derived nicotine-containing counterparts.

## 2. Materials and Methods

### 2.1. Procurement of Vape-Bars and E-Liquids

Three different TFN vape-bars and three different TFN e-liquids were analyzed in this study (Table 1). In addition to the six TFN vape-products analyzed, six different tobacco-derived nicotine-containing vape-bars were analyzed in this study. All vape-products (vape-bars and e-liquids) used in this study were either purchased from online vendors or local stores in the Rochester, NY area. All vape-bars and e-liquids used in this study have a salt nicotine concentration of 50 mg/mL or 5.0% nicotine by volume.

### 2.2. Acellular ROS Quantification within Generated Aerosols

**Figure 1:**
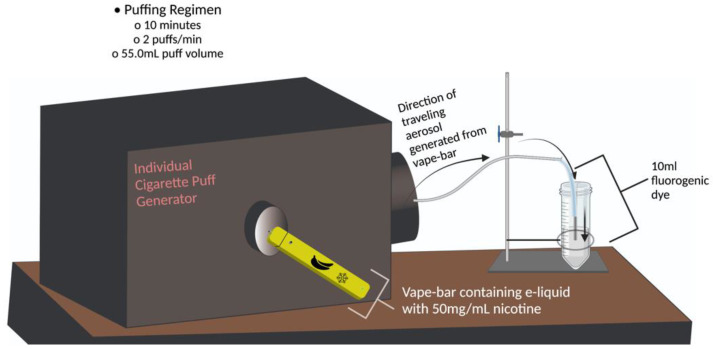
E-cigarette puff generator apparatus. The schematic shows the apparatus used to aerosolize each vape-bar and e-liquid included in this study. Once inserted into the Individual Cigarette Puff Generator, the component e-liquid within each vape bar was aerosolized for one individual puffing regimen; the generated aerosol was then exposed to 10 mL of fluorogenic dye during those ten minutes. One puffing regimen consisted of a vape-bar being aerosolized for 10 min and generating 20 total puffs, each puff lasting 3.0 s and having a volume of 55.0 mL. The entirety of the aerosolization process and the subsequent exposure of the generated aerosols to fluorogenic dye was done within a chemical fume hood. The pictogram was made using Adobe Illustrator and BioRender.

ROS levels within aerosols generated from all twelve vape-products were quantified via spectrofluorometry and in H_2_O_2_ equivalents. Aerosols from each individual TFN vape-product used in the study were generated using a Buxco Individual Cigarette Puff Generator (Data Sciences International (DSI), St. Paul, MN, USA) (Cat#601-2055-001) (Figure 1). Upon inserting the e-cigarette device into the central orifice apart of the adapter on the front side of the Puff Generator, the aerosol is generated and puffed by the mechanical part of the Puff Generator. Via tubing, the generated aerosols are then exposed to 10 mL of fluorogenic dye for a single puffing regimen at 1.5 L/min (Figure 1). One puffing regimen lasted for 10 min; 2 puffs/min, with each puff having a volume of 55.0 mL to simulate vaping topography parameters like puff volume, puff length, and puff duration This specific puffing regimen is identical to the puffing regimen used in our previous study analyzing acellular ROS levels with different flavored tobacco-derived nicotine-containing vape-bars and similar to the one used in another one of our previous studies examining acellular ROS levels generated by JUUL pods [4,14]. The fluorogenic dye used in the study was made from 0.01 N NaOH, 2′7′ dichlorofluorescein diacetate (H_2_DCF-DA) (EMD Biosciences, San Diego, CA, USA) (Cat#287810), phosphate (PO_4_) buffer, and horseradish peroxidase (Thermo Fisher Scientific, Waltham, MA, USA (Cat#31491). Each TFN e-liquid was aerosolized using a new, empty refillable JUUL Pod (OVNStech, Shenzhen, China) (Model: WO1 JUUL Pods) inserted into a JUUL device (JUUL Labs Inc., Washington, DC, USA) (Model: Rechargeable JUUL Device w/USB charger). Subsequently, this JUUL device was inserted into the Individual Cigarette Puff Generator.

Each vape-bar and JUUL Pod containing TFN e-liquid had undergone three separate puffing regimens to prepare three individual samples of 10 mL dye solution exposed to e-cigarette aerosols. For our negative control, filtered air was passed through fluorogenic dye using the previously mentioned puffing regimen and inserting a filter into the Individual Puff Generator instead of an e-cigarette. For our positive control, the smoke generated from a conventional cigarette (Kentucky Tobacco Research & Development Center in the University of Kentucky, Lexington, KY, USA) (Model Reference: 3R4F) was exposed to fluorogenic dye under the previously mentioned puffing regimen. To avoid cross-contamination, once a specific e-cigarette had undergone a single puffing regimen, the tubing connecting the Puff Generator to the 50 mL conical tube containing dye was rinsed with 70% ethanol and then double-distilled water (ddH_2_O). The tubing was also rinsed with 70% ethanol and ddH_2_O prior to generating puffs from a different e-cigarette model.

Subsequently, 0 μM, 10 μM, 15 μM, 20 μM, 30 μM, 40 μM, and 50 μM H_2_O_2_ standards were prepared using 30% H_2_O_2_ (Thermo Fischer Scientific, Waltham, MA, USA) (Cat#H323-500) and ddH_2_O. After aerosolizing each vape product and exposing its generated aerosols to three separate 10 mL samples of fluorogenic dye, each resulting fluorogenic dye sample and standard was placed in a 37 °C degree water bath (VWR International, Radnor, PA) (Model: 1228 Digital Water Bath) for fifteen minutes. After placing each sample and standard into the water bath, the resulting solutions were analyzed via fluorescence spectroscopy (Ex = 475 nm and Em = 535 nm). Readings were taken on a spectrofluorometer (Thermo Fischer Scientific, Waltham, MA, USA) (Model: FM109535) in fluorescence intensity units (FIU) and measured as H_2_O_2_ equivalents.

### 2.3. Statistical Analyses

One-way ANOVA and Tukey’s post-hoc test for multiple pairwise comparisons via GraphPad Prism Software version 8.1.1 was used to conduct statistical analyses of significance. Samples were run in triplicates. The results are shown as mean ± SEM with triplicate analyses. Data were considered to be statistically significant for *p* values < 0.05.

## 3. Results

### Differences in ROS Levels within Aerosols Generated by TFN Vape-Products and Tobacco-Derived Nicotine-Containing Vape-Products Vary with Flavor

**Figure 2:**
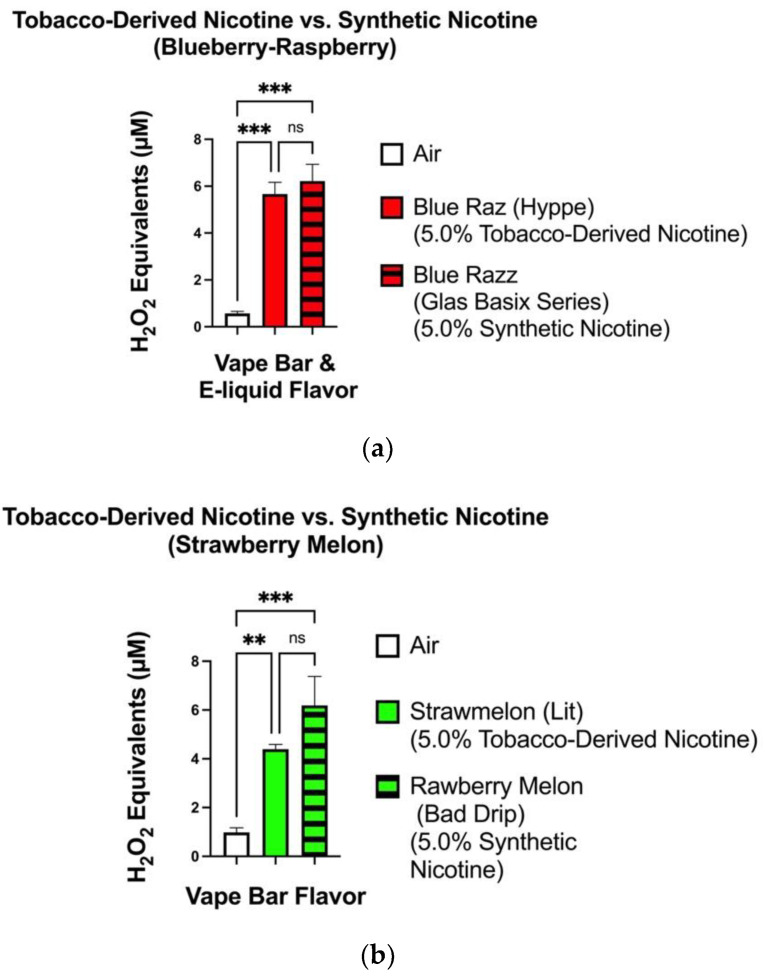
ROS levels within aerosols generated from blueberry-raspberry (**a**) and strawberry-melon (**b**) flavored tobacco-derived nicotine-containing and TFN vape-products. ROS levels within the generated aerosols from each individual TFN vape-product and tobacco-derived nicotine-containing vape-product was measured via spectrofluorometry and quantified as H_2_O_2_ equivalents. During analysis, the level of ROS generated from each individual vape-product was compared to the ROS generated from the filtered air control. Data are represented as mean ± SEM, and significance was determined by one-way ANOVA. ** *p* < 0.01 and *** *p* < 0.001 versus air controls. ns is abbreviated for “Non-Significant” versus air-controls (*p* > 0.05). Sample size (N) = 3–4.

For the blueberry-raspberry-flavored vape-products analyzed, the level of ROS generated from the Hyppe: Blue Raz (5.0% tobacco-derived nicotine) bar (4.92–6.61 μM) did not significantly differ from that generated from the GLAS Basix Blue Razz (5.0% synthetic nicotine) e-liquid (4.97–7.44 μM) (Figure 2a). Among the strawberry watermelon flavored vape-bars analyzed, the difference in acellular ROS levels in aerosols generated by the Bad Drip: Rawberry Melon (5.0% synthetic nicotine) vape-bar (3.82–7.48 μM) and Lit: Strawmelon (5.0% tobacco-derived nicotine) vape-bar (4.10–4.77 μM) was not significant (Figure 2b).

**Figure 3:**
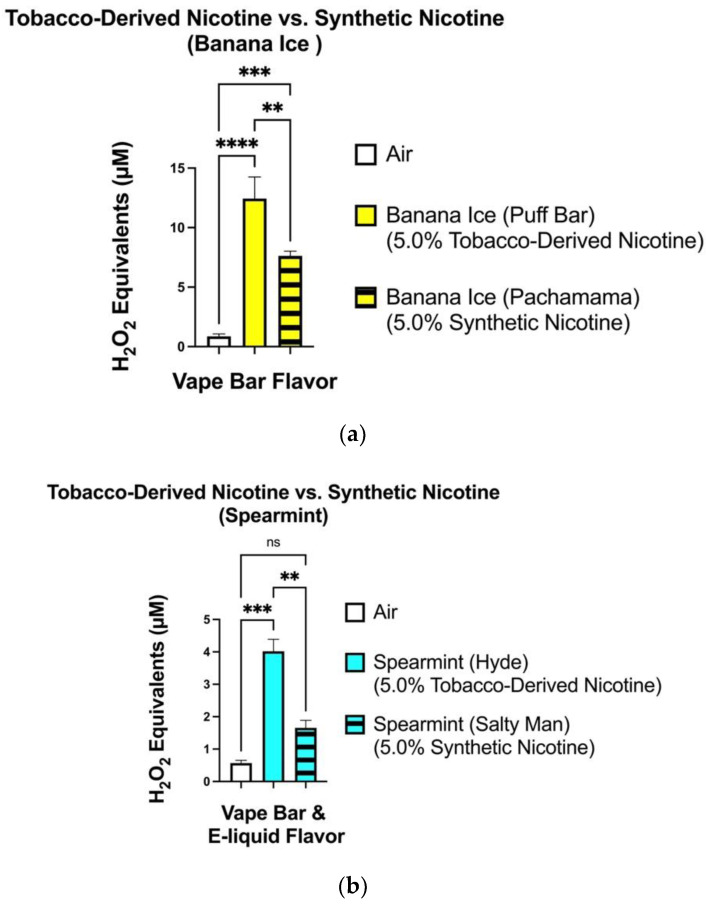
ROS generation among aerosols generated from banana ice (**a**) and spearmint (**b**) flavored TFN and tobacco-derived nicotine-containing vape-products. ROS levels within the generated aerosols from each individual minty/iced (cooled) flavored TFN and tobacco-derived nicotine-containing vape-product was measured via spectrofluorometry and quantified as H_2_O_2_ equivalents. During analysis, the level of ROS generated from each individual vape-bar was compared to the ROS generated from the filtered air control. Data are represented as mean ± SEM, and significance was determined by one-way ANOVA. ** *p* < 0.01, *** *p* < 0.001, and **** *p* < 0.0001 versus air controls. ns is abbreviated for “Non-Significant” versus air-controls (*p* > 0.05). Sample size (N) = 3–4.

Regarding minty/iced (cooled) flavored vape products, there appear to be significant differences in ROS levels generated between TFN vape products and their corresponding flavor-specific tobacco-derived nicotine counterparts (Figure 3). The level of ROS generated from the Pachamama: Banana Ice (5.0% synthetic nicotine) vape-bar (7.19–8.40 μM) differed significantly from that generated from the Puff Bar: Banana Ice (5.0% tobacco-derived nicotine) bar (9.69–15.87 μM) (Figure 3a). Similarly, the level of ROS generated from aerosolized Salty Mann: Spearmint (5.0% synthetic nicotine) e-liquid (1.33–2.11 μM) differed significantly from that generated from the Hyde: Spearmint (5.0% tobacco-derived nicotine) bar (3.28–4.50 μM) (Figure 3b).

**Figure 4:**
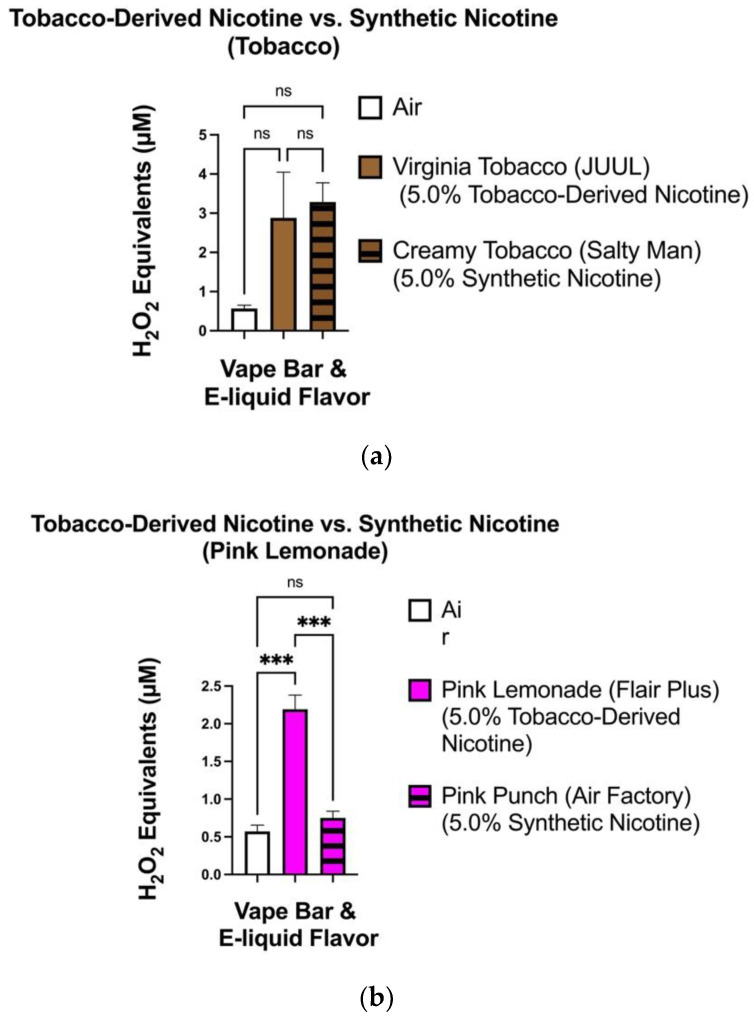
ROS generation among aerosols generated from tobacco (**a**) and drink flavored (**b**) TFN and tobacco-derived nicotine-containing vape-products. ROS levels within the generated aerosols from each individual tobacco and drink-flavored TFN e-liquid and tobacco-derived nicotine-containing vape-bar was measured via spectrofluorometry and quantified as H_2_O_2_ equivalents. During analysis, the level of ROS generated from each individual vape-bar was compared to the ROS generated from the filtered air control. Data are represented as mean ± SEM, and significance was determined by one-way ANOVA. *** *p* < 0.001 versus air controls. ns is abbreviated for “Non-Significant” versus air-controls (*p* > 0.05). Sample size (N) = 3–4.

**Figure 5:**
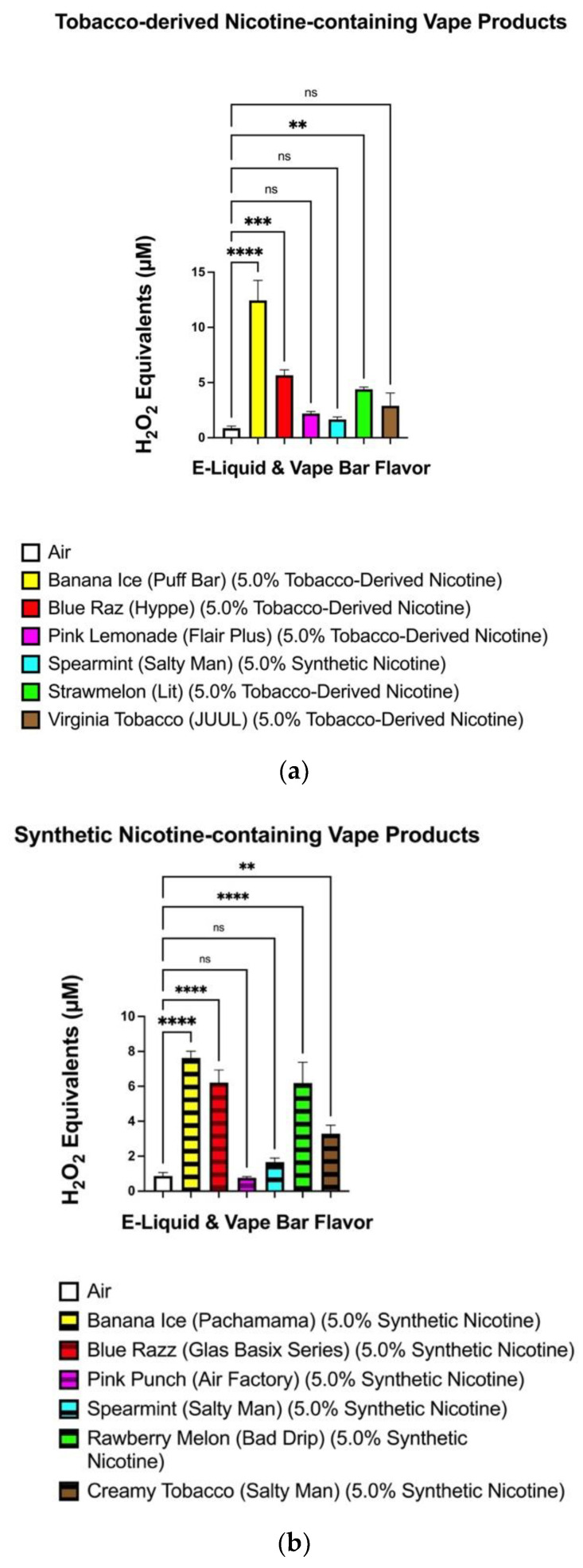
ROS generation among aerosols generated from tobacco-derived nicotine-containing (**a**) and TFN (**b**) vape-products ROS levels within the generated aerosols from each individual flavored TFN e-liquid and tobacco-derived nicotine-containing vape products were measured via spectrofluorometry and quantified as H_2_O_2_ equivalents. During analysis, the level of ROS generated from each individual vape-bar and e-liquid was compared to the ROS generated from the filtered air control. Data are represented as mean ± SEM, and significance was determined by one-way ANOVA. ** *p* < 0.01, *** *p* < 0.001, and **** *p* < 0.0001 versus air controls. ns is abbreviated for “Non-Significant” versus air-controls (*p* > 0.05). Sample size (N) = 3–4.

When comparing tobacco-flavored vape products, the level of ROS generated from the aerosolized Salty Man: Creamy Tobacco (5.0% synthetic nicotine) e-liquid (2.32–3.96 μM) did not significantly differ from that generated from the JUUL: Virginia Tobacco (5.0% tobacco-derived nicotine) bar (1.26–5.14 μM) (Figure 4a). However, regarding drink-flavored ENDS, the level of ROS generated from the Flair Plus: Pink Lemonade (5.0% tobacco-derived nicotine) bar (1.84–2.47 μM) was significantly different from that generated from the aerosolized Air Factory: Pink Punch (5.0% synthetic nicotine) e-liquid (0.61–0.92 μM) (Figure 4b). Regarding comparisons of the differences in ROS production between all flavors that had tobacco-derived nicotine and all flavors that had synthetic nicotine, we found particular flavored e-cigarettes containing Tobacco-derived nicotine generated significantly higher levels of ROS compared to the air control (0.21–1.59 μM) than their TFN-containing counterpart (Figure 5). More specifically, the difference in ROS levels generated by the Blue Razz, Strawberry Melon, and Tobacco-flavored vape-products containing tobacco-derived nicotine and the air control was higher than that between the corresponding flavored TFN vape-products and the air control (Figure 5).

## 4. Discussion

Our data suggest that the type of nicotine salt used in e-liquids and vape-bars, tobacco-derived or synthetic, plays a role in modulating ROS generation upon component e-liquid aerosolization. To further explain, significant differences in ROS generation were observed between TFN and tobacco-derived nicotine-containing vape-products containing drink and minty/iced flavoring. However, non-significant differences in ROS generation were observed between TFN and tobacco-derived nicotine-containing vape-products with fruity and tobacco flavoring. Our data suggest that flavoring agents used in e-cigarettes containing synthetic nicotine play a role in modulating ROS levels within generated aerosols. Our data also indicate that flavoring agents used in e-liquids affect acellular ROS generation from synthetic-nicotine-containing e-cigarettes and tobacco-derived nicotine-containing e-cigarettes of comparable flavors differently.

Similarly, the results of our study seem to concur with our previous study, the data of which suggested that flavoring agents used in tobacco-derived nicotine-containing vape-bars play a role in modulating ROS generation upon component e-liquid aerosolization [4]. Regarding the effects of nicotine content on ROS generation and oxidative stress, one study had found that nicotine increases oxidative stress in rat mesencephalic cells in a dose-dependent manner [15]. Another study found that aerosols from flavored e-cigarettes and e-liquids promoted oxidative stress in H292 lung epithelial cells as well as in the lungs of mice [16]. Additionally, one study found that ROS generated from e-cigarettes was highly dependent on the flavor of e-liquid used (fruity and tobacco) [5]. However, studies examining the differences in ROS generation within cellular and acellular systems due to the usage of tobacco-derived nicotine-containing and TFN vape- products are lacking. While previous studies have shown that voltage, flavoring, and nicotine concentration have a role in modulating e-cigarette generated ROS levels, the results of our study show that the type of nicotine salt used (synthetic or tobacco-derived) does as well [4,5,6].

Interestingly, we noticed that amongst the minty/cooled flavored vape-products analyzed (Spearmint and Banana Ice), the level of ROS generated by the synthetic-nicotine vape-product was significantly less than that generated by its flavor specific tobacco-derived nicotine-containing counterpart. Additionally, amongst the drink/beverage-flavored vape-products analyzed, the synthetic nicotine-containing vape product generated significantly less ROS than its tobacco-derived nicotine-containing counterpart. Synthetic nicotine lacks the impurities contained within tobacco-derived nicotine [11,17]. Vape products using synthetic nicotine lack tobacco specific nitrosamines (TSNAs), a carcinogen found in tobacco and tobacco-derived nicotine [11,17,18]. In our study, the differences in exogenous ROS between aerosols generated by TFN and tobacco-derived nicotine-containing vape-products with Pink Lemonade, Spearmint, and Banana-Ice flavoring may be due to the differences in impurities within each type of nicotine salt (tobacco-derived or synthetic) used. However, to determine whether the results observed for the Pink Punch Lemonade, Spearmint, and Banana Ice flavored ENDS are due to differences in the level of impurities within the salt nicotine used, e-cigarette screening via inductively coupled plasma mass spectrometry (ICP-MS) is needed.

Regarding the limitations of this study, due to there being very few companies that manufacture both TFN and tobacco-derived nicotine-containing vape-products, we could not control for the e-cigarette brand in our pairwise comparisons between TFN products and their flavor specific tobacco-derived nicotine-containing counterparts, as well as differences between enantiomers or stereoisomers (R-nicotine vs. S-nicotine) of nicotine in both the products. Many vendors which utilize synthetic nicotine in their vape products either never sold e-cigarettes using tobacco-derived nicotine or stopped selling them entirely due to the cost-burden associated with submitting PMTAs and lack of public interests, and confirming the validity of synthetic vs. natural nicotine. One study has shown that even amongst e-cigarettes of the same flavor, ROS levels within generated aerosols vary by brand [4]. Future studies examining the differences in ROS levels generated by TFN vape products and their flavor-specific tobacco-derived nicotine-containing counterparts of the same company are needed, as well as cellular studies.

## 5. Conclusions

Our data suggest that TFN tobacco flavors and fruit flavors are more toxic in terms of ROS generation than menthol/ice and drink/beverage flavored products using TFN. In other words, beverage flavor and minty/iced (cool) flavored TFN products generate significantly less ROS than their corresponding flavor-specific tobacco-derived nicotine-containing counterparts. Our study provides insight into how interactions between flavoring agents and salt-nicotine used in e-cigarettes impact ROS levels generated by TFN e-cigarettes differently than e-cigarettes using tobacco-derived nicotine.

## Figures and Tables

**Table 1:** Tobacco-derived and tobacco-free nicotine ENDS used in this study.

Company	Flavor	Nicotine Concentration (mg/mL)	Nicotine Salt-Type
Air Factory	Pink Punch ( Pink Punch Lemonade)	50.0	TFN
Bad Drip	Rawberry Melon	50.0	TFN
Flair Plus	Pink Lemonade	50.0	Tobacco-Derived
Glas (BASIX Series)	Blue Razz	50.0	TFN
Hyppe	Blue Raz	50.0	Tobacco-Derived
Hyde	Spearmint	50.0	Tobacco-Derived
JUUL	Virginia Tobacco	50.0	Tobacco-Derived
Lit	Strawmelon	50.0	Tobacco-Derived
Pachamama	Banana Ice	50.0	TFN
Puff Bar	Banana Ice	50.0	Tobacco-Derived
Salty Man	Creamy Tobacco	50.0	TFN
Salty Man	Spearmint	50.0	TFN

## Data Availability

We declare that we have provided all the data in figures.
